# Clinical SARS-CoV-2 Kinetic Profiles Are Dependent on the Viral Strain and Host Vaccination Status

**DOI:** 10.21203/rs.3.rs-1978917/v1

**Published:** 2022-09-20

**Authors:** Manjula Gunawardana, John Cortez, Jessica Breslin, Simon Webster, Nash Rochman, Peter Anton, Marc Baum

## Abstract

The SARS-CoV-2 infection kinetics in a real-world, clinical setting represent a knowledge gap in understanding the underlying COVID-19 pathogenesis. There are scant reports on the dynamics describing the two principal components of the viral life cycle, namely the rapid proliferation and slower clearance phases. Here, we present results from an ongoing workplace clinical surveillance study where two vaccinated participants became infected with SARS-CoV-2 Omicron variant (BA.1. lineage). The subjects were followed longitudinally at high temporal resolution allowing the kinetics of both viral phases to be characterized. The viral doubling times in the proliferation phase (3.3-3.5 h) and maximum measured viral loads were similar to those observed for unvaccinated individuals infected with an earlier SARS-CoV-2 strain. However, the clearance phase was much shorter in the current study and unexpectedly displayed a multimodal profile. Longitudinal whole genome SARS-CoV-2 sequencing identified a stable mutation that arose in one of the participants over the 2-week period of positivity. Our small study provides a rare insight into the clinical SARS-CoV-2 dynamics holding significance to public health measures and the biology underlying COVID-19.

## Background

A comprehensive understanding of SARS-CoV-2 dynamics throughout the entire viral growth cycle is key to describing the underlying disease pathogenesis and is needed to inform effective public health measures and clinical management policies. Since March 23, 2020, we have been conducting a continuous, ongoing workplace clinical study involving the longitudinal and intensive characterization of COVID-19 prevalence and incidence.[1] This intensely sampled observational study has enabled participants who developed COVID-19 to be identified in the early stages of exponential viral growth (*i.e.*, proliferation phase), and allowed them to be observed longitudinally *via* serial measurements with high temporal resolution[2]. In our previous study, we followed unvaccinated participants who developed COVID-19 in late 2020-early 2021, including the measurement of viral RNA copy numbers by RT-qPCR analysis of nasal swab samples. We successfully characterized the viral kinetics of the rapid proliferation and slow clearance phases. The median clinical SARS-CoV-2 doubling time, $t_d$, during the growth phase was calculated as 3.1 h (range 2.8-5.2 h), and the time between the highest measured viral loads ($C_{max}$) and a negative test result exceeded 40 days in all participants, in one case extending to 90 days.

Here, we followed two participants –a male (subject 35) and a female (subject 56), both aged 58-67 years– who developed COVID-19 independently between December 27, 2021 (subject 35) and January 3, 2022 (subject 56). Both subjects were vaccinated at the time they became infected with SARS-CoV-2 (see **Fig. 1** caption), with no health-concerning conditions nor taking any immunomodulating medication. They initially developed symptoms within hours of their first positive RT-qPCR tests: subject 35, *ca*. 4-5 hours post-sample collection; subject 56, *ca*. 12-15 hours prior to sample collection. The last positive SARS-CoV-2 test for subject 35 was 11 days post-diagnosis with symptom duration being 6 days. For subject 56, the last positive test was 10 days post-diagnosis with resolution of symptoms in 12 days.

Immediately following their first positive result, nasal swab samples were collected every 12 hours for viral load analysis by RT-qPCR. At many timepoints, paired nasal samples were collected for SARS-CoV-2 whole genome sequencing. The results are summarized in **Fig. 1**, and capture the complete viral growth life cycles.

The $t_d$ and $C_{max}$ values were comparable to those observed in our previous study[2] (**Fig. 1F**), but the clearance phase was much shorter, with both participants being SARS-CoV-2 RNA positive for less than 2 weeks (**Fig. 1A** and **Fig 1D**). The observed rapid clearance could be ascribed to the viral strain, the vaccination status of the participants, or a combination of host-virus factors. It has been suggested that amplification and detection of the short sequences targeted by primers used in the clinical RT-qPCR tests may measure SARS-CoV-2 RNA fragments rather than infectious virus, thereby biasing the length of the observed clearance phase. Our results contradict this rationale as we previously measured SARS-CoV-2 RNA in COVID-19 patients for up to 90 days[2], while the time from $C_{max}$ to undetectable was less than 1 week in the current study using the same methods.

We also measured a nonmonotonic trend in nasal viral load over time in both participants, an observation that was more pronounced for subject 35 (**Fig. 1A**). Interestingly, the doubling time in the second phase of viral growth was considerably longer than the first (5.1 *versus* 3.3 h, **Fig. 1B** and **1C**).

Paired nasal swab samples from the above participants also were prepared for SARS-CoV-2 whole genome sequencing (subject 35, 18 samples; subject 56, 14 samples). Sequence analysis identified the viral strain as Omicron BA.1. The initial viral sequences in both individuals were nearly identical and could be distinguished by only a single residue, S R158S observed in subject 56, but not subject 35, and may be relevant to antibody escape[3]. Participants had no physical contact and acquired the infection from independent sources. In addition, we analyzed the sequences longitudinally to determine if any viral mutations emerged in the 14-day window of host-supported viral replication. After accounting for likely sequencing errors, we identified a single mutation that emerged over the period of observation (between 7 AM and 7 PM on January 4, 2022) and persisted. This mutation occurred in subject 35, and matches the residue in subject 56, namely ORF1ab S546P.

Our results engender a number of caveats. First, only a small study population was observed, predicated by the difficulty in prospectively identifying asymptomatic individuals who just became infected with SARS-CoV-2, and subsequently following those individuals longitudinally at high sampling frequency. Second, the concept of doubling time is derived from the biology underlying cellular division in microorganisms such as bacteria, and does not strictly apply to viruses. However, the parameter is useful in describing the kinetics underlying the exponential expansion of viral populations.

The current report represents a timely and impactful contribution towards elucidating the kinetics of the SARS-CoV-2 Omicron variant (BA.1. lineage) proliferation with a doubling time of 3.3-3.5 hours, and time to highest viral concentration under 3 days with a documented clearance time under clinical conditions in middle-aged participants. Our results provide a real-world context to interpreting human challenge study data obtained under controlled conditions, using ancestral viral strains and young, healthy adults[4].

## Methods

**Ethics statement.** All human research under OCIS-05, "Longitudinal Characterization of COVID-19 Prevalence and Incidence in a Small Working Institution with Both Public Health and Diagnostic Aims", was approved by Aspire IRB (Aspire Study # 1281548) and conducted according to the Declaration of Helsinki. All study participants provided written informed consent or assent.

**Clinical study design.** The workplace SARS-CoV-2 surveillance clinical study was initiated by the Oak Crest Institute of Science (Oak Crest, https://www.oak-crest.org/), a small nonprofit academic science research organization located in Monrovia, CA, on 23 March, 2020, has been running without interruptions, and is ongoing at the time of writing. The study design has been described in detail elsewhere[1,2].

**Calculation of SARS-CoV-2 doubling time**. The *in vivo* SARS-CoV-2 doubling time ($t_d$) during the exponential growth phase (*i.e.*, proliferation phase) was calculated according to methods described elsewhere[2].

**Whole genome SARS-CoV-2 sequencing**. Nasal swab samples for sequencing were preserved in RNA shield buffer (300 µL, R1200-125, Zymo Research, CA), frozen at -80°C, and stored/transported at -80°C. Samples were prepared for whole genome SARS-CoV-2 sequencing using the tailed amplicon method[5] at the University of Minnesota Genomics Center (UMGC, Minneapolis, MN). Briefly, RNA was extracted using QIAamp Viral RNA Mini kit (52904, Qiagen, MD) and cDNA and amplicon libraries were generated according to the published ARTIC v3 protocol[5]. The sequencing method employed 95 PCR primer pairs to tile the SARS-CoV-2 genome with overlapping amplicons in a total of four multiplexed amplification reactions to achieve virtually complete genomic assembly.

**SARS-CoV-2 sequence analysis**. Genomes sampled across multiple timepoints for two individuals were aligned to reference sequence Wuhan-Hu-1, NC_045512.2 using MAFFT[6]. All gaps were adjusted to respect the open reading frames of the reference sequence as previously described[7]. Only one substitution in a noncoding region which does not precede ORF1ab or follow ORF10 (A28271T, conserved in all samples) was observed and noncoding regions were removed. Only one conserved insertion was identified, consistent with S 214 insEPE[8] in both individuals as well as several nonsynonymous substitutions that enabled the identification of all variants as Omicron BA.1. The genomes from the two individuals can be distinguished by a single residue, S R158S, observed in individual 56 but not 35 across all samples for each individual. Additionally, several substitutions and small deletions that were not temporally conserved and were attributed to sequencing errors were observed (S11 gtc→gtt, S del151-2, S H954Q, S K969N, S F981L, E I9T, and 7b T40I).

**Data analysis**. Data sets were analyzed using GraphPad Prism (version 9.4.0; GraphPad Software, Inc., La Jolla, CA).

## Data Availability

All other data supporting the findings of this manuscript are available from the corresponding author (MMB) upon reasonable request.

## Declarations

#### Acknowledgements

The study was funded entirely through discretional, institutional funds. N.D.R. is supported by the Intramural Research Program of the NIH (National Library of Medicine). The authors gratefully acknowledge this support and Oak Crest Institute of Science's dedication to providing a safe work environment for its employees. We also gratefully acknowledge all study participants for donating their time and the valuable clinical specimens.

#### Author contributions

MG, PAA, and MMB conceptualized and designed the study. MG, NDR, and MMB performed the data analyses. MG, JMC, JB, SW, and MMB conducted the study and/or collected data. MG, NDR, PAA, and MMB interpreted the data. MMB wrote the first draft. JB and NDR critically revised the article. MG and MMB supervised the project. All authors read and provided final approval of the version to be published.

#### Competing interests

The authors have no competing interests.

### Figures

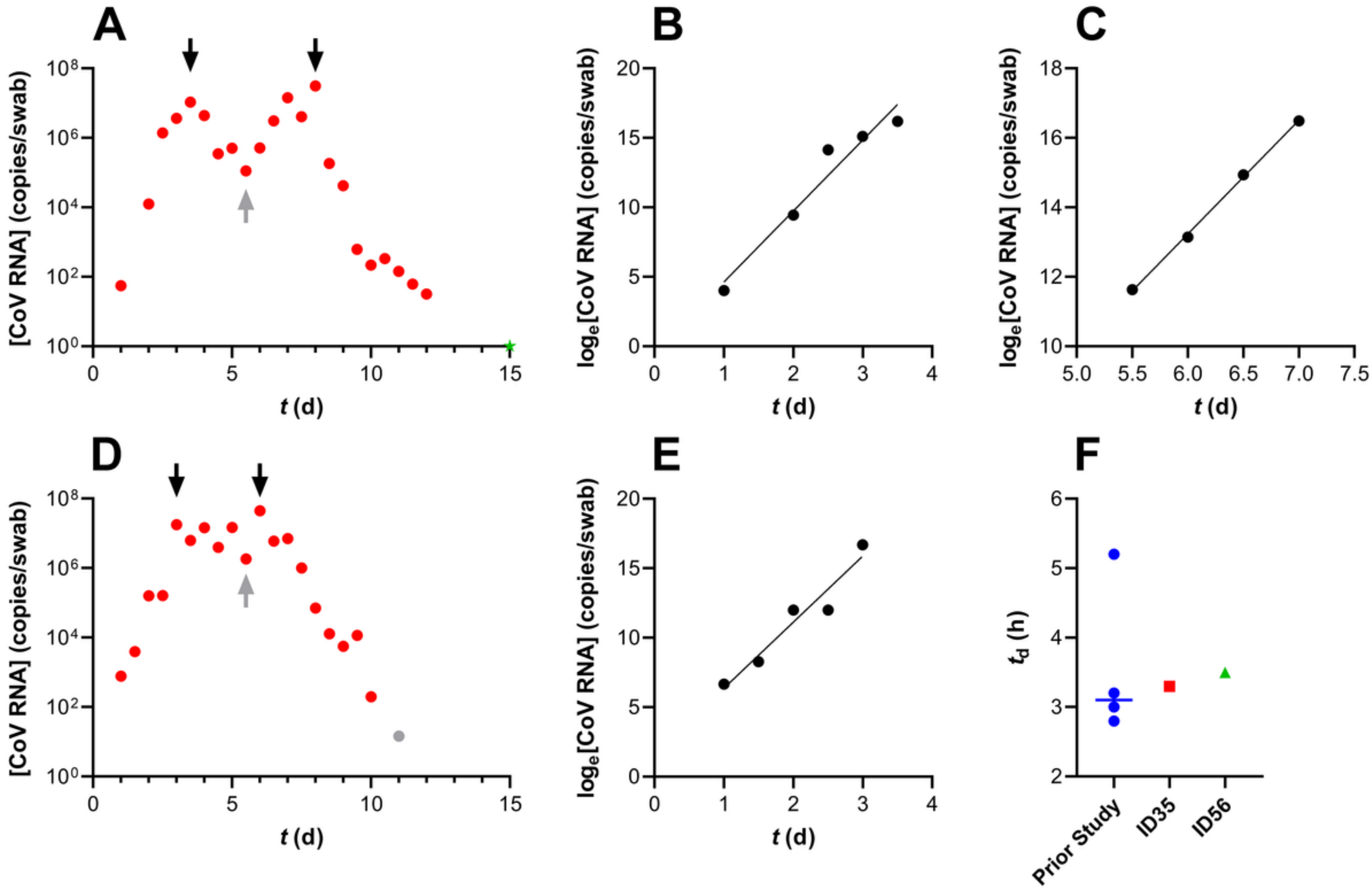

#### Figure 1

**High resolution SARS-CoV-2 (Omicron BA.1 . strain) viral dynamics in two clinical study participants with mild COVID-19**. **A** and **D**, The viral load kinetics for subjects 35 and 56, respectively, display similar profiles. Red, positive; grey, inconclusive; green, negative; black arrows identify viral load maxima (*Cmax*), while grey arrows identify viral load minima. **B** and **C**, The log-transformed viral loads plotted against time for subject 35 were used to calculate the proliferation phase (*td*, 3.3 h; *R2*, 0.947) and mid-infection (*td*, 5.1 h; *R2*, 0.999) doubling times, respectively. **E**, The log-transformed viral loads plotted against time for subject 56 were used to calculate the proliferation phase doubling time (*td*, 3.5 h; *R2*, 0.934). **F**, Comparison of the viral proliferation doubling times from the current study with our previous study2 involving unvaccinated participants infected with an earlier SARS-CoV-2 strain. Participant SARS-CoV-2 vaccination history: subject 35, Pfizer-BioNTech (03/19/21 and 04/09/21), Moderna (11/13/21); subject 56, J&J-Janssen (04/01/21), Pfizer-BioNTech (10/27/21).
